# Overview of the Epidemiology of Pancreatic Cancer Focusing on the JACC Study

**DOI:** 10.2188/jea.15.S157

**Published:** 2005-08-18

**Authors:** Dongmei Qiu, Michiko Kurosawa, Yingsong Lin, Yutaka Inaba, Tsuyoshi Matsuba, Shogo Kikuchi, Kiyoko Yagyu, Yutaka Motohashi, Akiko Tamakoshi

**Affiliations:** 1 Department of Epidemiology and Environmental Health, Juntendo University School of Medicine.; 2 Department of Public health, Aichi Medical University School of Medicine.; 3 Department of Public Health, Akita University school of Medicine.; 4 Department of Preventive Medicine/ Biostatistics and Medical Decision Making, Nagoya University Graduate School of Medicine.

**Keywords:** Pancreatic Neoplasms, Risk Factors, the JACC Study

## Abstract

BACKGROUND: The objective of this article was to overview the epidemiology of pancreatic cancer. We summarize the results of the Japan Collaborative Cohort Study (JACC Study) and some previous studies.

METHODS: References were mainly in a Medline search through Pub Med database. In addition, 3 papers about the JACC Study were quoted.

RESULTS : In the JACC Study, the standardized mortality ratio of pancreatic cancer was 0.97 in females and 0.84 in males. Diabetes mellitus (DM) has increased the risk for pancreatic cancer in many studies. In the JACC Study, DM had a risk for pancreatic cancer in males (hazard ratio = 2.12). Cigarette smoking has been associated with pancreatic cancer in many studies. In the JACC Study, the hazard ratio for current smokers was 1.6 in males, and 1.7 in females. The ratio was 3.3 who smoked 40+ cigarettes/day in males. In the JACC Study, alcohol intake was not associated with pancreatic cancer. These results are consistent with the other studies. Coffee consumption has not been associated with pancreatic cancer in many studies. In the JACC Study, the hazard ratio significantly increased to 3.19 among men who consumed 4+ cups of coffee per day. The relationships between diet /nutrition and pancreatic cancer are not clear in many studies.

CONCLUSION: The relation between smoking and pancreatic cancer is most consistently described. A further analysis of the relationships between family history, hormonal factors in females, dietary and nutritional factors, obesity, physical activity and pancreatic cancer is necessary.

Although pancreatic cancer is a relatively rare cancer, it is associated with high fatality. The etiology of pancreatic cancer remains largely unknown. There are no screening tests for early detection of pancreatic cancer. Many epidemiologic studies have been conducted to identify the risk factors for pancreatic cancer. The objective of this article was to overview the epidemiology of pancreatic cancer.

We referred the results of focusing on the (Japan Collaborative Cohort Study) JACC Study and reviewed previous studies. The reason for focusing on the JACC Study is that it is ongoing large scale population-based cohort study of a total of over 110,000 Japanese followed up.

References were mainly in a Medline search through Pub Med database which have been published in English since 1994, by using keywords of pancreatic cancer, risk factor, and epidemiologic study. Reports were selected on the basis of the best available evidence for each factor discussed. Additionally, 3 papers about the JACC Study: (Mortality in the JACC Study till 1999),^1^ (A prospective cohort study of cigarette smoking and pancreatic cancer in Japan),^2^ and (Risk of pancreatic cancer in relation to alcohol drinking, coffee consumption and medical history: findings from the Japan collaborative cohort study for evaluation of cancer risk)^3^ were quoted. In the JACC Study, the strength of associations was examined using hazard ratios (HRs) derived from the Cox proportional hazards model. The HRs were adjusted for potentially confounding factors. Risk factors data, such as life style and medical history, were obtained with self-administered questionnaire at baseline.

## Incidence and Mortality of Pancreatic Cancer

Pancreatic cancer has demonstrated increasing trends in both incidence and mortality during the past four decades in Japan,^4^ although its morbidity and mortality in Japan had been among the lowest in the world.^5^^,^^6^ It is the 5th leading cause of death from cancer in males and the 6th leading cause in females in Japan according to the latest statistics in Japan.^7^ The age-adjusted mortality rate from pancreatic cancer increased 3.0-fold in males and 2.9-fold in females in Japan between 1960 and 2002.^7^^,^^8^ The risk of pancreatic cancer in blacks was approximately 50% higher than that in whites among the general population.^9^ High incidence of pancreatic caner was found in New Zealand Maoris, especially in Maoris females.^10^ In the United States high incidence was occurred among Caucasian and African- American males and females.^11^

During approximately 12 years of follow up in the JACC Study, 161 males and 156 females deaths from pancreatic cancer occurred out of a total of 46,465 males and 64,327 females.^1^ The standardized mortality ratio (SMR) of pancreatic cancer in females in the JACC Study was similar to that in Japan nationwide (0.97), whereas in males it was somewhat lower than that in Japan nationwide (0.84).^1^ This low SMR of pancreatic cancer in males might partly come from the selection of investigated subjects in the JACC Study.^1^ Because some of cohort subjects in the JACC Study were chosen out of participants in routine medical checkups or other kinds of screening, of which was regarded relating to slightly healthier lifestyles.^1^

## Age and Sex

The risk of pancreatic cancer increases with advancing age. Nearly 80% of the patients with pancreatic cancer were between 60 and 80 years of age.^12^ The similar founding was reflected in the JACC Study, which observed 79% of deaths aged 60 or older.^1^ Pancreatic cancer occurs more frequently in males than in females.^9^^,^^10^^,^^13^ The age-adjusted death rate of pancreatic cancer in males was approximately 1.7-fold higher than that in females in 2002 in Japan.^7^

Hormonal factors related to pancreatic cancer in females were observed by analysis of menarche, menopause and productive history in previous studies.^14^^-^^18^ Although the mechanism of hormonal factors for pancreatic cancer remains unclear, pancreatic cancer is at least in part association with hormonal levels in females.^14^ It can be expected to verify the association between pancreatic cancer and hormonal factor in the JACC Study, which investigated 64,327 females over approximately 12-year follow up.^1^

## Medical Conditions

### (1) Diabetes mellitus (DM)

A number of epidemiologic studies have shown that there is a complex relationship between DM and pancreatic cancer.^13^^,^^19^^-^^23^ In a large prospective study in the US, Calle et al.^19^ found that the risk of pancreatic cancer mortality was significantly increased not only among subjects with a history of diabetes, but also among diabetics during the second and third years of follow-up (HR = 2.05, 95% confidence interval [CI]: 1.56-2.69] (Table 1). Compared with the controls, a 70% or 50% higher risk for pancreatic cancer was observed in subjects who were diagnosed with diabetes 5-9 years prior to the diagnosis of pancreatic cancer and among subjects who were diagnosed with diabetes at least 10 years prior to the diagnosis of pancreatic cancer, respectively, in a case-control study in the US.^20^ (Table1) A meta-analysis of pancreatic cancer studies found that individuals with DM for a duration of at least 5 years had an increased risk for pancreatic cancer (HR = 2.0, 95% CI: 1.2-3.2).^24^ Studies conducted in Japan, Sweden, and the US^19^^,^^21^^,^^25^ also found that patients with DM had an increased risk for pancreatic cancer (Table 1).

**Table 1.** Relation of medical conditions to the risk of pancreatic cancer.

Author	Year	Place	Study		Sex	Item	Risk ratio	95% CI	Adjusted factors
JACC Study Lin et al.^28^	1988-1997	Japan	Cohort		Male	History of diabetes mellitus			age and cigarette smoking in pack-years
						No	1.00	(reference)	
						Yes	2.12	1.19-3.77	

					Female	History of gallstone/cholecystitis			
						No	1.00	(reference)	
						Yes	2.51	1.41-4.46	

Calle et al.^17^	1982-1994	US	Cohort		Both	A history of diabetes			age, sex, race, smoking status, body mass index, family history of pancreatic cancer, and education
						No	1.00	(reference)	
						Yes	1.48	1.30-1.68	

					Male	No	1.00	(reference)	
						Yes	1.49	1.25-1.77	

					Female	No	1.00	(reference)	
						Yes	1.51	1.24-1.85	

						Follow-up time (years)			
						No	1.00	(reference)	
						1-3	2.05	1.56-2.69	
						4-6	1.44	1.11-1.86	
						7-9	1.28	0.97-1.68	
						10-12	1.38	1.08-1.77	

Silverman^18^	1986-1989	US	Case-control		Both	Interval between onset of diabetes and diagnosis of cancer			age at diagnosis/interview, race, gender, area, cigarette smoking, alcohol consumption, body mass index, and calories from food
						No diabetes	1.0	(reference)	
						5-9 years	1.7	1.0-2.9	
						≧10 years	1.5	1.01-2.2	
						Interval between onset of cholecystectomy and diagnosis of cancer			
						No cholecystectomy	1.0	(reference)	
						20+ years	1.7	1.0-3.0	

La Vecchia et al.^20^	1983-1992	Italy	Case-control	Both		A history of diabetes			age and sex
						No	1.0	(reference)	
						Yes	2.1	1.5-2.9	

Gapstur et al.^27^	1967-1995	US	Cohort		Both	Postload plasma glucose level			age, race, categories of postload plasma glucose concen-tration, cigarette smoking status, and quartiles of body mass index
						-119 mg/dL	1.00	(reference)	
						120-159 mg/dL	1.65	1.05-2.60	
						160-199 mg/dL	1.60	0.95-2.70	
						200+ mg/dL	2.15	1.22-3.80	

Johansen et al.^33^	1977-1992	Denmark	Cohort		Both	Diagnosis of gall stones			age, sex, and calendar year
						No	1.00	(reference)	
						Yes	1.33	1.1-1.6	

Chow et al.^29^	1997-1993	Denmark	Cohort		Both	cholecystectomy patients			age and sex
						Years of follow-up 5+ year	1.30	1.1-1.6	

Schernhammer et al.^31^	1982-1998	US	Cohort		Both	History of gallstones or cholecystectomy			age in months, follow-up cycle, history of diabetes, smoking status, nonvigorous physical activity in metabolic equivalents per week, in quintiles, cohort baseline, and baseline body mass index
						No	1.00	(reference)	
						Yes	1.11	0.78-1.56	

CI: confidence interval.

On the other hand, La Vecchia et al.^22^ suggested that DM was an initial symptom of pancreatic cancer rather than a cause of pancreatic cancer based on a case-control study in Italy, because the risk declined as the number of years after the diagnosis of DM increased, although the subjects with DM as a whole had a significantly elevated risk (HR = 2.1; 95% CI: 1.5-2.9). (Table 1) A case-control study conducted in northern Italy also obtained similar results.^23^ Gullo et al. found that there was no association between DM and pancreatic cancer among patients with diabetes of 3 or more year duration, and presumed that diabetes was caused by the pancreatic cancer and that the increasing prevalence of diabetes in patients with pancreatic cancer was mainly due to diabetes of recent onset.^26^ In a case-control study performed in hospitals in New Zealand, it was concluded that diabetes might be an epiphenomenon of pancreatic cancer, rather than a risk factor for pancreatic cancer.^27^ Furthermore, in a nested case-control study on familial pancreatic cancer in the US, the authors concluded that DM was not a risk factor for pancreatic cancer.^28^

In order to avoid misclassification of DM, Gapstur et al.^29^ studied the postload plasma glucose level instead of self-reported diabetes as the diagnostic criterion in a cohort study conducted over a mean period of 25 years. A significant dose-response association was found between the postload plasma glucose level and pancreatic cancer mortality. Compared with the subjects with postload plasma glucose level of 119mg/dL or less, the HR of pancreatic cancer mortality was 1.65 (95% CI: 1.05-2.60) for those with 120-159 mg/dL, 1.60 (95% CI: 0.95-2.70) for those with 160-199mg/dL, and 2.15 (95% CI: 1.22 -3.80) for those with 200mg/dL or higher (Table 1). This association was stronger in males than in females. These results suggested that abnormal glucose metabolism plays an important role in the development of pancreatic cancer, and that diabetes was likely to be a risk factor for this tumor.^29^

In the JACC Study, an increased risk for pancreatic cancer in subjects with a history of DM was found in males and females, only in male was significant (HR = 2.12, 95% CI: 1.19-3.77).^3^ (Table 1) The positive association between DM and pancreatic cancer from this unique large cohort study in Japan was consistent with most of previous studies.^19^^-^^23^ The increasing death rates of pancreatic cancer may partly explain by the increasing of DM in Japan. Overall, DM might be both an initial manifestation of pancreatic cancer as well as a risk factor of this malignant tumor. Further investigations are required to explore the relationship between long-standing DM and pancreatic cancer.

### (2) Cholecystectomy/Gallstones or Cholecystitis

A positive association between pancreatic cancer and cholecystectomy was observed.^20^^,^^30^ Chow reported an increased risk of pancreatic cancer for cholecystectomy 5 or more years prior to the diagnosis of pancreatic cancer^30^ in a cohort study. (Table 1) A case-control study also showed a 70% excess risk among subjects who underwent cholecystectomy 20 or more years prior to the cancer diagnosis.^20^ (Table 1) Although some studies found no evidence for an association between cholecystectomy and pancreatic cancer,^21^^,^^31^^,^^32^ the causality was supported by experiment study: cholecystectomy increased circulating levels of cholecystokinin, which is a promoter of pancreatic carcinogenesis in rodents.^33^ In the JACC Study, there was no significant relationship was observed between cholecystectomy and pancreatic cancer, but the risk of death from pancreatic cancer was significantly increased in females with gallstones or cholecystitis (HR = 2.51, 95% CI: 1.41-4.46).^3^ (Table 1) This result was consistent with that in a cohort study in Denmark.^34^ (Table 1)

### (3) Others

Previous studies suggested that chronic pancreatitis plays an important role in the development of pancreatic cancer,^13^^,^^35^^-^^39^ but the clinical relevance of a causal relationship between chronic pancreatitis and pancreatic cancer was limited.^39^ Although hereditary pancreatitis resembles other types of pancreatitis, the age of onset of hereditary pancreatitis is much earlier. The risk of pancreatic cancer of hereditary pancreatitis was 50-60 times higher than expected compared with the background population.^10^

Two case-control studies suggested that immune function in relation to allergy might play a role in the etiology of pancreatic cancer.^40^^,^^41^ No association was found between regular aspirin use and pancreatic cancer in a case-control study in the US.^42^ In laboratory studies, aspirin inhibited the cell growth of four pancreatic cancer cell lines, and aspirin use may be a possible therapy for prevention of pancreatic cancer.^43^ In a recent cohort study, however, the risk of pancreatic cancer was significantly elevated with extending periods of regular aspirin use in females.^44^

The association between pancreatic cancer and chronic pancreatitis, hereditary pancreatitis, allergy or aspirin use can not verify in the JACC Study. Further investigations into the mechanism of these observed associations are warranted.

## Family history

Family aggregation of pancreatic cancer has been found. Genetic factor played an important role of the etiology of pancreatic cancer. Among those subjects with first-degree relatives with pancreatic cancer, the risk for pancreatic cancer ranged from 1.2 to 32.0.^20^^,^^25^^,^^28^^,^^45^^-^^47^ In particular, the risk increased as the number of first-degree relatives with familial pancreatic cancer increased.^46^ Additionally, the risk of pancreatic cancer mortality was significantly increased in subjects with a family history of uterine cancer and breast cancer.^48^ These findings will be verified in the JACC Study.

## Smoking, Alcohol, and Coffee Consumption

### (1) Smoking

Table 2 shows cigarette smoking for the risk of pancreatic cancer in the JACC Study and other epidemiologic studies. According to the results of the JACC Study, upon analysis of mortality to the end of 1997, cigarette smoking was associated with pancreatic cancer mortality.^2^ Regarding the JACC Study, compared with non-smokers, the HR for current smokers was 1.6 (95% CI: 0.95-2.6) in males, and 1.7 (95% CI: 0.85- 3.4) in females.^2^ The HR of death from pancreatic cancer according to the number of cigarettes smoked per day was 3.3 (95% CI: 1.38-8.1) among subjects who smoked 40 cigarettes or more per day in males.^2^ The age at which one started to smoke, number of years of smoking and number of pack-years were not significantly associated with an increased risk for death from pancreatic cancer.^2^

**Table 2.** Cigarette smoking for the risk of pancreatic cancer.

Author	Year	Place	Study	Sex	Item	Risk ratio	95% CI	Adjusted factors
JACC study Lin Y et al.^48^	1988-1997	Japan	Cohort	Male	Never	1.0	(reference)	age, BMI, history of DM, gallbladder diseases
				Male	Ex-smoker	1.1	0.61-1.9	
				Male	Current smoker	1.6	0.95-2.6	

				Female	Never	1.0	(reference)	
				Female	Ex-smoker	1.8	0.67-5.0	
				Female	Current smoker	1.7	0.85-3.4	

JACC study Lin Y et al.^48^	1988-1997	Japan	Cohort		Cigarettes/day (Current smokers)			
				Male	Never	1.0	(reference)	
				Male	1-19	1.6	0.91-2.9	
				Male	20-39	1.3	0.74-2.4	
				Male	40+	3.3	1.38-8.1	

Fuch CS et al.^54^	1980-1996 1986-1998	US	2 cohorts		Pack years for current smokers			age in 2-year intervals, sex, BMI, and history of DM
				both	Never	1.0	(reference)	
				both	1-10	1.3	0.3-5.4	
				both	11-25	2.7	1.4-5.1	
				both	26-50	2.8	1.8-4.4	
				both	50+	2.1	1.2-3.8	

Stolzenberg-Solomon RZ et al.^79^	1985-1997	Finland	Cohort (smokers)		Pack years			age,intervention(alpha-tocopherol and beta carotine supplement)
				Male	<22	1.00	(reference)	
				Male	22-31	1.18	0.69-2.03	
				Male	32-39	1.23	0.71-2.12	
				Male	30-49	1.26	0.75-2.13	
				Male	49+	1.66	1.02-2.72	

CI: confidence interval.

BMI: body mass index.

DM: diabetes mellitus.

In many epidemiologic studies, cigarette smoking has been reported to be associated with increased risk for pancreatic cancer. A study of the cancer registry in Sweden showed that smoking increased the risk of pancreatic cancer.^47^ The relationship between smoking and pancreatic cancer has been studied in case-control^13^^,^^20^^,^^23^^,^^25^^,^^49^^,^^50^ and cohort studies.^51^^-^^59^ These results show that smokers have an increased risk for pancreatic cancer. Most studies showed that heavy smokers have a higher risk for pancreatic cancer than light smokers. The dose-response relationship was observed in several case-control studied.^20^^,^^50^^,^^60^ Although the risk for pancreatic cancer did not change after smoking cessation for more than 2 years prior to the interview (odds ratio [OR] = 1.4, 95% CI: 1.1-1.9],^20^ after 15 years from ceasing smoking, the risk for pancreatic cancer dropped to the level of a lifetime non-smoker regardless of the lifetime smoking amount.^21^

As for the previous cohort studies,^51^^-^^59^ the HR for pancreatic cancer ranged from 1.3 to 3.9 among current smokers, and a significant dose-response relationship was observed in 4 studies.^54^^,^^56^^,^^59^^,^^61^ A significant, positive trend in risk for pancreatic cancer as the number of pack-years of smoking increased was observed in another cohort study,^54^ especially in current smokers and when the analysis was confined to cigarette smoking within the past 15 years. Additionally, the results of a cohort study of male smokers showed that pack-years over 49 number was associated with an increased risk for pancreatic cancer compared with pack-year less than 22 number (HR = 1.66, 95% CI: 1.02-2.72).^61^

The JACC Study and most studies showed that current smokers have a higher risk than nonsmokers. The dose-response relationship was observed in the JACC Study and the other studies, however it was not strong in these studies.

### (2) Alcohol consumption

Table 3 shows alcohol consumption for the risk of pancreatic cancer in the JACC Study and other epidemiologic studies. According to the results of the JACC Study, upon analysis of mortality to the end of 1997, alcohol intake was not associated with pancreatic cancer mortality.^3^ A case-control study in the US showed that black females who drank heavily had an increased risk for pancreatic cancer (OR = 2.5, 95% CI: 1.02-5.9).^20^ Inoue et al.^25^ conducted a case-control study in Japan and showed that former drinkers had an increased risk for pancreatic cancer. In two cohort studies conducted in the US, alcohol intake was not associated with an increased risk for pancreatic cancer (30+ grams of alcohol/day versus none; HR=1.00, 95% CI: 0.57- 1.76).^62^ These results are consistent with those of the JACC Study.

**Table 3.** Alcohol consumption for the risk of pancreatic cancer.

Author	Year	Place	Study	Sex	Item	Risk ratio	95% CI	Adjusted factors
JACC study Lin Y et al.^28^	1988-1997	Japan	Cohort		Daily amount(g)			
				Male	Non-drinker	1.00	(reference)	age, cigarette smoking in pack year
				Male	Ex-drinker	0.74	0.30-1.82	
				Male	Current drinkers 0-29(g)	1.16	0.66-2.04	
				Male	Current drinkers 30-59(g)	1.07	0.56-2.06	
				Male	Current drinkers 60+(g)	0.98	0.39-2.46	

Silverman et al.^18^	1986-1989	US	Case-control		Number of drinks/week*			
				Female	Never drank	1.0	(reference)	age, area, cigarette smoking, gallbladder disease, DM
				Female	1-8	1.1	0.5-2.2	
				Female	8-21	1.8	0.8-4.0*	1 drink=l.5oz.of hard liquor or 12 oz. of beer or 4 oz. of wine
				Female	21+	2.5	1.02-5.9	

Inoue M. et al.^23^	1988-1999	Japan	Case-control		Alcohol drinking			age, family history of pancreatic cancer, history of DM, regular physical exercise, bowel habit, raw vegetable intake
				both	Never	1.0	(reference)	
				both	Ever	0.8	0.57-1.12	
				both	Ever-former	3.7	2.28-6.00	
				both	Ever-current	0.5	0.34-0.73	

Michaud DS et al.^62^	1980-1996 1986-1998	US	2 cohorts		Alcohol intake (g/day)			
				both	0	1.00	(reference)	pack years of smoking, BMI, history of DM, cholecy-sectomy, energy intake and period
				both	0.1-1.4	0.78	0.47-1.30	
				both	1.5-4.9	1.15	0.78-1.69	
				both	5.0-29.9	1.00	0.69-1.44	
				both	30+	1.00	0.57-1.76	

CI: confidence interval.

BMI: body mass index.

DM: diabetes mellitus.

### (3) Coffee consumption

Table 4 shows coffee consumption for the risk of pancreatic cancer in the JACC Study and other epidemiologic studies. According to the results of the JACC Study, upon analysis of the data obtained through the end of 1997, coffee intake was not associated with pancreatic cancer mortality.^3^ However, the HR significantly increased to 3.19 (95% CI: 1.22-8.35) among men who consumed a large amount of coffee, i.e., 4+ cups of coffee per day.^3^

**Table 4.** Coffee consumption for the risk of pancreatic cancer.

Author	Year	Place	Study	Sex	Item	Risk ratio	95% CI	Adjusted factors
JACC study Lin Y et al.^28^	1988-1997	Japan	Cohort	Male	Nondrinker	1.00	(reference)	
				Male	1-2cups/month	0.74	0.37-1.49	age, cigarette smoking in pack year
				Male	1-4cups/week	0.58	0.32-1.08	
				Male	1cup/day	0.59	0.26-1.33	
				Male	2-3cups/day	0.75	0.36-1.59	
				Male	4+cups/day	3.19	1.22-8.35	

Partanen et al.^63^	1984-1990	Finland	Case-control	both	None/occasional	1.00	(reference)	sex, birth year, smoking
				both	1-3cups/day	0.83	0.50-1.60	
				both	4-6cups/day	0.96	0.59-1.56	
				both	6+cups/day	0.71	0.41-1.20	

Stensvold et al.^64^	1977-1990	Norwey	Cohort		Cups per day			
				Female	-2	1.0	(reference)	age
				Female	3-4	-		
				Female	5-6	1.2		
				Female	7+	1.0		

Michaud DS et al.^62^	1980-1996 1986-1998	US	2 cohorts	both	None	1.00	(reference)	pack years of smoking, BMI, history of DM, cholecysectomy, energy intake and period
				both	-1/day	0.94	0.65-1.36	
				both	1/day	0.60	0.38-0.94	
				both	2-3/day	0.88	0.65-1.21	
				both	3+/day	0.62	0.27-1.43	

CI: confidence interval.

BMI: body mass index.

DM: diabetes mellitus.

Coffee has been studied as a cause of pancreatic cancer after the warning that coffee was related to pancreatic cancer. There was no association between coffee consumption and the risk of pancreatic cancer in a case-control study in Finland,^63^ nor in a cohort study in Norwegian men and women.^64^ Coffee intake was not associated with an increased risk for pancreatic cancer in two cohort studies conducted in the US (HR = 0.62, 95% CI: 0.27-1.43).^62^ In these two cohort studies, there were also no statistically significant associations between intake of tea, intake of decaffeinated coffee, or total caffeine intake and pancreatic cancer.^62^ Most previous studies did not find an association between coffee consumption and pancreatic cancer.

## Dietary and Nutritional Factors

Many studies on the relationship between dietary intake and pancreatic cancer have been conducted.^13^ However, there are often difficulties related to ascertaining accurate dietary information from these patients. Because pancreatic cancer has a high fatality rate and due to the difficulty of dietary research, the relationship between diet and nutrition is not clear.

Many case-control studies suggested that higher cholesterol intake increases the risk of pancreatic cancer.^16^^,^^60^ There are few reports, intake of grilled red meat might be a risk factor for pancreatic cancer in a case-control study.^65^ In a cohort study, in male smokers the intake of butter and the intake of saturated fat increased the risk of pancreatic cancer.^66^ In a cohort of the US women, the intake of total fat, different types of fat, cholesterol, total meat, red meat, and eggs were not associated with pancreatic cancer.^67^ The relation between pancreatic cancer and intake of cholesterol and intake of fat are not clear.

In case-control studies, high caloric intake increased the risk of pancreatic cancer.^20^^,^^68^^,^^69^ The interaction between body mass index (BMI) and energy intake suggests the importance of energy balance in pancreatic carcinogenesis.^20^ However, in a cohort study of male smokers, high caloric intake reduced the risk of pancreatic cancer.^66^ The results of the relation between pancreatic cancer and caloric intake are not consistence. When the relationship between pancreatic cancer and caloric intake is analyzed, it is necessary to combine the factors of obesity and physical activities.

As to preventive factors, consumption of vegetables was protective against pancreatic cancer in a case-control study in Japan.^25^ In a cohort of male smokers, dietary folate intake^61^ and high intake of carbohydrates reduced the risk of pancreatic cancer.^66^ However, in a cohort of US women, carbohydrate intake was not associated with pancreatic cancer.^70^ A detailed analyses of the relationship between nutrition including total calorie intake, cholesterol intake, etc., and pancreatic cancer is necessary.

## Obesity and Physical Activity

Most studies have shown a high incidence of pancreatic cancer among obese persons, and it may suggest that as the BMI increases, the risk of pancreatic cancer increases.^59^^,^^68^^,^^71^^,^^72^ Total physical activity was inversely associated with pancreatic cancer risk.^72^ A detailed analysis of the relationship between nutrition and pancreatic cancer is necessary to combine the factors of diet, obesity and physical activities.

## Conclusion

The incidence and mortality of pancreatic cancer have been increasing during the past four decades in Japan. The standardized mortality ratio of pancreatic cancer in females in the JACC Study was similar to that in Japan, whereas it was little lower in males. Rates increase with an advancing age in the JACC Study. It was similar to previous studies. The relation between smoking and pancreatic cancer is most consistently described. Histories of DM and gallstones / cholecystitis were the risk of pancreatic cancer in the JACC Study. However, many studies have shown that there is a complex relationship between DM and pancreatic cancer. Further investigations will be required for DM and other medical conditions. Alcohol was not associated with and increased risk. Most previous studies did not find the association between the coffee consumption and the pancreatic cancer, although a large amount of coffee consumption increased the risk in the JACC Study. A further analysis of the relationships between family history, hormonal factors in females, dietary and nutritional factors, obesity, physical activity and pancreatic cancer is necessary.

## MEMBER LIST OF THE JACC STUDY GROUP

The present investigators involved, with the co-authorship of this paper, in the JACC Study and their affiliations are as follows: Dr. Akiko Tamakoshi (present chairman of the study group), Nagoya University Graduate School of Medicine; Dr. Mitsuru Mori, Sapporo Medical University School of Medicine; Dr. Yutaka Motohashi, Akita University School of Medicine; Dr. Ichiro Tsuji, Tohoku University Graduate School of Medicine; Dr. Yosikazu Nakamura, Jichi Medical School; Dr. Hiroyasu Iso, Institute of Community Medicine, University of Tsukuba; Dr. Haruo Mikami, Chiba Cancer Center; Dr. Yutaka Inaba, Juntendo University School of Medicine; Dr. Yoshiharu Hoshiyama, Showa University School of Medicine; Dr. Hiroshi Suzuki, Niigata University School of Medicine; Dr. Hiroyuki Shimizu, Gifu University School of Medicine; Dr. Hideaki Toyoshima, Nagoya University Graduate School of Medicine; Dr. Shinkan Tokudome, Nagoya City University Graduate School of Medical Science; Dr. Yoshinori Ito, Fujita Health University School of Health Sciences; Dr. Shuji Hashimoto, Fujita Health University School of Medicine; Dr. Shogo Kikuchi, Aichi Medical University School of Medicine; Dr. Akio Koizumi, Graduate School of Medicine and Faculty of Medicine, Kyoto University; Dr. Takashi Kawamura, Kyoto University Center for Student Health; Dr. Yoshiyuki Watanabe, Kyoto Prefectural University of Medicine Graduate School of Medical Science; Dr. Tsuneharu Miki, Kyoto Prefectural University of Medicine Graduate School of Medical Science; Dr. Chigusa Date, Faculty of Human Environmental Sciences, Mukogawa Women’s University ; Dr. Kiyomi Sakata, Wakayama Medical University; Dr. Takayuki Nose, Tottori University Faculty of Medicine; Dr. Norihiko Hayakawa, Research Institute for Radiation Biology and Medicine, Hiroshima University; Dr. Takesumi Yoshimura, Institute of Industrial Ecological Sciences, University of Occupational and Environmental Health, Japan; Dr. Akira Shibata, Kurume University School of Medicine; Dr. Naoyuki Okamoto, Kanagawa Cancer Center; Dr. Hideo Shio, Moriyama Municipal Hospital; Dr. Yoshiyuki Ohno, Asahi Rosai Hospital; Dr. Tomoyuki Kitagawa, Cancer Institute of the Japanese Foundation for Cancer Research; Dr. Toshio Kuroki, Gifu University; and Dr. Kazuo Tajima, Aichi Cancer Center Research Institute.
